# A Rare Case of Continuous Type Splenogonadal Fusion in a Young Male with Primary Infertility

**DOI:** 10.1155/2014/796761

**Published:** 2014-05-14

**Authors:** Santosh Kumar, Kumar Jayant, Swati Agrawal, Kalpesh Mahesh Parmar, Shrawan Kumar Singh

**Affiliations:** Department of Urology, PGIMER, Chandigarh 160012, India

## Abstract

Splenogonadal fusion is a rare developmental anomaly in which an abnormal connection between splenic tissue and gonads or mesonephric derivatives is present. Here we present a case of young man with the complaint of primary infertility for 3 years. On evaluation (USG and MRI abdomen and pelvis), his right scrotal testis was atrophied and left intra-abdominal undescended testis. On laparoscopic assessment, a mass was seen on the left side due to continuous type of splenogonadal fusion for which excision and left orchidectomy were done. Postoperative period was uneventful and he was discharged under satisfactory condition. Splenogonadal fusion is a rare entity and it is commonly mistaken for testicular tumour. It should be considered in the differential diagnosis of testicular masses especially when there are associated congenital anomalies and preoperative laparoscopic assessment, should be done to avoid unnecessary radical surgery.

## 1. Introduction

Splenogonadal fusion is a very uncommon developmental anomaly arising due to abnormal connection between developing splenic tissue from dorsal mesogastrium and gonadal or mesonephric derivatives. Such rare anomaly usually takes place during the 5th to 8th week of gestation, that is, before gonadal descent starts and presents as mass. The product fusion commonly diagnosed as a testicular mass of unknown origin in association with other congenital deformities [1].

## 2. Case Report

**Figure 1:**
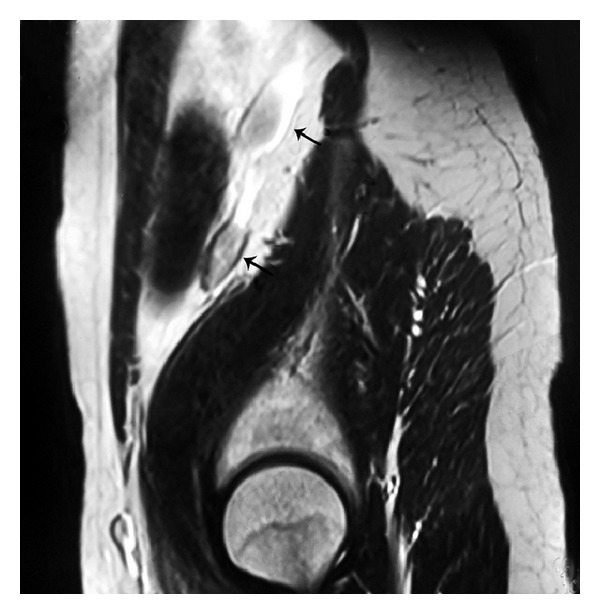
MRI T2, sagittal cuts showing 2 oblong masses in the left retroperitoneum.

**Figure 2:**
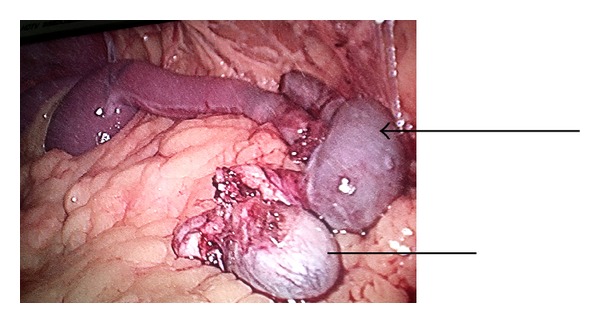
Laparoscopic assessment picture showing splenogonadal fusion (splenic portion marked with arrow-line and testicular portion marked with line only).

**Figure 3:**
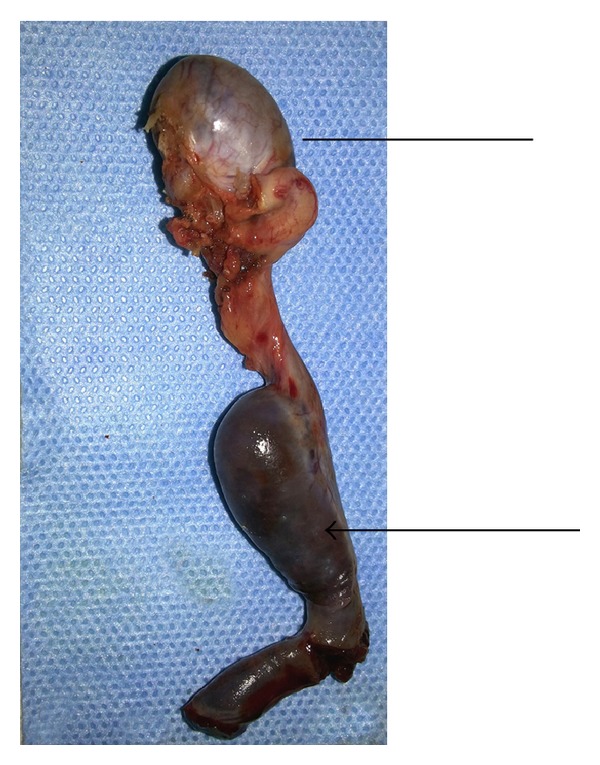
Resected specimen of the left orchidectomy with fused splenic tissue (splenic portion marked with arrow-line and testicular portion marked with line only).

**Figure 4:**
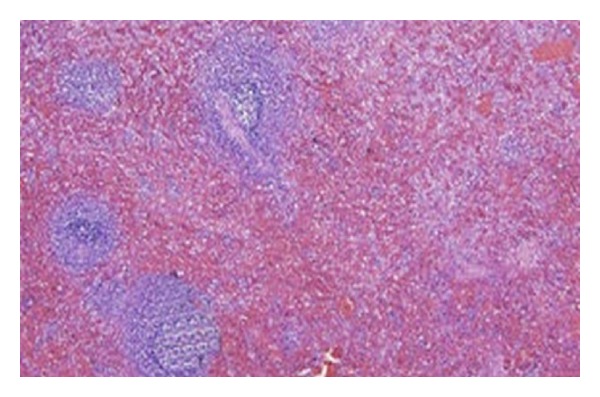
Microscopic picture confirming spleen showing white and red pulp in mass. (H&E stain ×400 magnification).

A 25-year-old male patient presented in urology clinic with chief complaint of primary infertility of a 3-year duration. General physical examination was normal. On local examination, patient's right atrophic testis was situated in scrotum and of size 1.5 cm × 1.5 cm. On the other hand, there was empty scrotum on left side which was suggestive of undescended testes. There were no associated congenital anomalies seen. Routine blood investigations, renal function test, and liver function tests were normal. The levels of tumor markers as AFP and beta-hCG were 5 kIU/L and 2.5 kIU/L which were within normal limits. Semen analysis revealed azoospermia. Hormonal study revealed primary testicular failure with elevated gonadotropin levels. Ultrasonography (USG) revealed absence of left sided testis and epididymis in the scrotum and inguinal canal. MRI reported two oblong masses each 2.5 × 1 cm in the left retroperitoneal region anterior and lateral to psoas muscle at L3 to S1 level with atrophied left seminal vesicles (Figure 1). Patient was taken for laparoscopic assessment and was found to have left sided testicular mass with no lymph nodal enlargement (Figure 2). On careful examination, it was suggestive for splenogonadal fusion anomaly for which he underwent left orchidectomy (Figure 3). The specimen was sent for histopathology which showed the tumor mass to be composed of fibrous capsule with red and white pulp within it suggestive of spleen and there was no evidence of spermatogenesis in the testicular portion of resected specimen (Figure 4). He fared well in postoperative period and was discharged on postoperative day 3. Later, he was followed up on out-patient basis and was doing well.

## 3. Discussion

The first description of splenogonadal fusion was made by Bostroem in 1883, and a detailed report by Pommer followed in 1889. There are few other names existing for this disorder in literature as ectopic scrotal spleen or testicular splenic fusion. In a review of 30 cases,  Putschar and Manion categorised splenogonadal fusion into continuous and discontinuous types depending on the anatomical continuity between the principal spleen and the gonad [2]. The continuous type is characterized by connection of the spleen and the gonad by a cord of splenic or fibrous tissue. Rarely, beads of splenic tissue are interspersed throughout the fibrous cord. In the discontinuous type, ectopic splenic tissue is attached to the gonad but has no connection to the normally located spleen [3]. Accessory spleen is usually found within the tunica vaginalis and is closely attached to the gonad, although a distinct capsule is present. Splenogonadal fusion is assumed to occur between 5 and 8 weeks of gestation, before the beginning of gonadal descent. Its cause remains unclear, but two theories predominate. First, slight inflammation of the peritoneal surfaces over the spleen and gonadal ridge can produce partial fusion of the two organs, while the second has postulated that a retroperitoneal pathway for splenic anlage cells may allow contact with the gonadal anlage [4]. This malformation has predominantly been reported in males, with a male to female ratio of 15 : 1. Most cases are usually seen before the age of 20 and more than half of them were seen before the age of 10 years. Although the left testis is predominantly involved, cases of right splenogonadal fusion have also been reported [5]. Splenogonadal fusion is so rare that it almost never gives any impression of its existence in the nature and thus is rarely diagnosed preoperatively. Mostly, it is incidentally diagnosed as an asymptomatic mass while exploring inguinal region for some other reasons as for cryptorchidism, a hernia, or a hydrocele. Presentation is varied from scrotal mass to testicular torsion, most common presentation being testicular swelling. Another important presentation is acute onset painful scrotal lump secondary to testicular torsion or involvement of the ectopic splenic tissue as a corollary to varied presentation of disorders as in malaria, leukemia, infectious mononucleosis, and traumatic rupture of the ectopic spleen.

Continuous type of splenogonadal fusion has been associated with many anomalies, including cryptorchidism, which has been found in approximately 33% of the cases of splenogonadal fusion. Other associated anomalies include cardiac defects, limb bud abnormalities also called peromelia (severe congenital deformity of limbs which closely mimics that of thalidomide embryopathy), micrognathia, hypoglossia, craniosynostosis, spina bifida, palatine, and anorectal and rare syndromic anomalies. Discontinuous type is usually not related to any congenital anomaly though few cases of associated cardiac defects are reported in the literature. Although the case we presented here was of continuous type of splenogonadal fusion, there was no other associated congenital anomaly, except cryptorchidism and infertility [6].

Techniques of diagnostic imaging are available if there is a clinical suspicion of splenogonadal fusion. The most reliable investigation to confirm its diagnosis is preoperative imaging through 99 m Tc-sulfur colloid liver spleen scan, which detects accessory spleen. Recently, Doppler ultrasonography of the scrotum of patients with a palpable testicular mass has been used to diagnose preoperatively few cases of splenogonadal fusion by visualizing a hypervascular mass on the upper pole of testis and comparing it with patient's own normally located spleen [7, 8]. Such use of this modality again depends on high index of suspicion in cases of splenogonadal fusion presenting as cryptorchidism, but it may be of value in patients presenting with acute scrotal lump. Unaware of the nature of the left scrotal masses, many surgeons have sacrificed an intact testis because they lacked a proper preoperative diagnosis. Surgeons ignorant of the nature of the scrotal mass and fearing malignant degeneration have performed radical orchidectomy on salvageable testes [9]. In our cases, as the patient had primary gonadal failure and laparoscopic assessment was suggestive of benign testicular mass due to splenogonadal fusion with atrophic testis, so simple excision of mass was done. This highlights the importance of laparoscopic assessment for the nature of the mass, which saves the patient from undergoing a radical surgery.

## 4. Conclusion

Splenogonadal fusion is a very uncommon disorder and due to its presentation as an asymptomatic testicular mass it can easily be misdiagnosed as malignancy though involved structure is of normal characteristics which suggests its benign nature. It must be considered as an important differential diagnosis of testicular masses, more so if it is associated with congenital anomalies. Preoperative laparoscopic assessment may prevent unnecessary radical orchidectomy but, in cases in which salvage is done for cryptorchidism, surveillance for detection of any future occurrence of malignancy is recommended.
